# RUNX1 Is an Independent Prognostic Marker Involved in Immune Infiltration and Therapeutic Responses in Clear Cell Renal Cell Carcinoma Patients Through Cluster Regulation With RUNX2

**DOI:** 10.1002/iid3.70242

**Published:** 2025-08-14

**Authors:** Jingang Jian, Jun Zhang, Hongbo Xu, He Wang, Jianquan Hou

**Affiliations:** ^1^ Department of Urology The Fourth Affiliated Hospital of Soochow University Suzhou China; ^2^ Department of Urology The First Affiliated Hospital of Soochow University Suzhou China

**Keywords:** ccRCC, cluster regulation, immune infiltration, prognosis, RUNX1, RUNX2, therapeutic response

## Abstract

**Background:**

Immunomodulation is an important factor in clear cell renal cell carcinoma (ccRCC) carcinogenesis and the treatment outcome of ccRCC patients. Understanding of the roles of the immune‐related Runt‐related transcription factor (RUNX) family genes in ccRCC remains controversial, and the mechanisms underlying their cluster regulation and immunomodulatory effects still need to be further investigated.

**Methods:**

The “limma” package was used for differential gene expression analysis of TCGA and GEO data, and the “clusterProfiler” package was used for GO and KEGG enrichment analyses of coexpressed and differentially expressed genes. The “MCPCounter” algorithm and “CIBERSORT” algorithm were used to explore the correlation between gene expression and immune cell infiltration, and prognostic models were constructed using the “rms” package.

**Results:**

RUNX1/2/3 showed elevated expression in ccRCC, with high expression of RUNX1/2 correlating with advanced clinicopathological characteristics and poor prognosis. Functional enrichment analysis and immune infiltration scores indicated that RUNX1/2 are extensively involved in the formation of the tumor immunosuppressive microenvironment. Analyses of immune checkpoint data and immunotherapy responses suggest that RUNX1 may inhibit immunotherapy effects. Multi‐Cox regression models confirmed that RUNX1 can be used as an independent prognostic indicator. In addition, an efficient clinical prognostic model can be constructed based on RUNX1 combined with several clinicopathological characteristics (AUC = 0.872).

**Conclusion:**

Our study reveals the potential value of RUNX1/2 in ccRCC development, prognosis, and immune microenvironment regulation. RUNX1 is a promising biomarker for the tumor immunotherapy response. Furthermore, the RUNX1‐based prognostic model has the prospect of serving as a better predictive model for clinical ccRCC.

## 1 Introduction

Renal cell carcinoma (RCC), one of the most common malignant urinary tumors, has gradually increased in incidence over the past 20 years and accounts for 3%–5% of all new cancers [1]. Recent epidemiological studies suggest that this rise in RCC incidence may, in part, be attributed to the emergence of new favorable subsets of cancers of unknown primary (CUP), including RCC CUP. This newly recognized clinical entity is managed similarly to RCC and may contribute to the observed increase in RCC incidence [2]. Clear cell RCC (ccRCC) is the most common and aggressive type of RCC, with more than 90,000 cancer‐related deaths worldwide each year [3]. Compared with early‐stage localized ccRCC, which can be effectively cured by surgical resection, the treatment of metastatic ccRCC (mccRCC) is more difficult and has a worse prognosis [4]. Therefore, it is critical to clarify the etiology and mechanisms of ccRCC progression, to discover prognostic markers that can be used clinically and to develop more reliable prognostic prediction models. ccRCC is an “immunotherapy responsive” tumor, and tumor‐infiltrating immune cells have an important role in ccRCC progression and patient prognosis [5, 6]. In recent years, with the approval of immune checkpoint inhibitors (ICIs), such as Nivolumab (anti‐PD‐1) and Ipilimumab (anti‐CTLA‐4), for the clinical treatment of mccRCC patients, the regulation of the tumor immune microenvironment (TME) and the corresponding immunotherapy have been extensively studied in ccRCC [7]. However, from a therapeutic standpoint, tyrosine kinase inhibitor (TKI) monotherapy remains a valid first‐line treatment option for a significant proportion of patients who are not candidates for immunotherapy. The results of the STAR trial directly apply to this patient group. For other patients, although TKI is not typically used as a first‐line therapy, it may be considered as a second‐line monotherapy. While treatment interruption may be a reasonable consideration for these patients, caution is warranted, as they generally experience shorter progression‐free survival compared to those receiving first‐line TKI treatment [8]. A growing number of studies have demonstrated that the immunomodulatory capacity of the TME is essential for improving immunotherapy for ccRCC patients and shows potential prognostic value [9, 10].

The runt‐related transcription factor (RUNX) family, as essential transcription factors, is involved in the regulation of various developmental processes, such as proliferation, differentiation, and apoptosis [11]. It has three tissue‐specific members expressed in mammals: RUNX1, RUNX2, and RUNX3. RUNX1 is located on chromosome 21, RUNX2 on chromosome 6, and RUNX3 on chromosome 1. Increasingly, studies have shown that RUNX genes exert complex and bidirectional regulatory roles in multiple hematologic and solid tumors, including but not limited to leukemia and ovarian, skin, colorectal, prostate, gastric, and breast cancers [12, 13]. For example, RUNX1 promotes tumor progression in colorectal and skin cancers, while exhibiting tumor suppressive activity in breast and prostate cancers [14, 15]. Similarly, RUNX2 and RUNX3 exhibit similar bidirectional functions in different tumors [16, 17, 18]. Recent studies have shown that RUNX genes also play an important role in ccRCC. RUNX1 and RUNX2 are highly expressed in ccRCC, and reduced methylation levels of RUNX1 are significantly associated with poor prognosis [19, 20]. However, the biological mechanisms and prognostic implications of the members of the RUNX family in ccRCC are still debated. Moreover, RUNX cluster regulation in ccRCC has rarely been reported. Therefore, understanding the regulatory mechanisms of RUNX genes in ccRCC and their impact on the TME may be elucidate potential strategies to treat mccRCC. In addition, evaluating the potential of RUNX family members as reliable prognostic markers holds significant importance for predicting outcomes in patients with ccRCC.

In this study, we clarified the differential RUNX gene transcriptional and proteomic expression by analyzing gene expression profiles and tissue immunohistochemistry and revealed its regulatory role in the TME in ccRCC by enrichment analysis, immune cell infiltration assessment, and immunotherapy responsiveness studies. We also combined RUNX expression levels and several key clinicopathological characteristics to build a RUNX1‐based nomogram to predict the prognosis of ccRCC patients. In conclusion, our findings are the first to suggest that RUNX1 and RUNX2 activity in ccRCC may contribute to the formation of an immunosuppressive microenvironment in ccRCC tumors through a cluster regulatory mechanism. RUNX1 and RUNX2 expression are significantly associated with the levels of multiple immune checkpoint genes. Among them, the expression of RUNX1 may have an important suggestive role in immunotherapy. In addition, we provided a more effective predictive model for the prognosis of ccRCC.

## 2 Methods

### 2.1 Data Source and Expression Analyses of RUNX1/2/3

The mRNA levels of RUNX1/2/3 in 33 cancers were analyzed using the Tumor Immune Estimation Resource (TIMER) database with The Cancer Genome Atlas (TCGA) data (https://cistrome. shinyapps. io/timer/) [21]. The mRNA expression data of 535 cases of clear cell renal cell carcinoma (ccRCC) and 72 cases of normal kidney tissues were extracted from the TCGA data through the “TCGAbiolinks” package (version 3.6.2), and the expression was normalized by log2(FPKM + 1) [22]. The GSE53757 (tumor/normal = 72/72) and GSE76351 (tumor/normal = 12/12) microarray data sets were extracted from the GEO database, and the data were log2(x + 1) normalized [23]. The “limma” package (version 3.40.6) was used to analyze the differential gene expression between the ccRCC tumor and normal groups in TCGA and GEO data [24]. For the expression profile data set, we performed multiple linear regression by the lmFit function and further processed the data by the eBayes function to finally obtain the significance of differences for each gene (|logFC | > 0.585, FDR < 0.05). The transcription factor data set was extracted from HumanTFDB (http://bioinfo.life.hust.edu.cn/). The expression of RUNX1/2/3 in ccRCC subtypes was explored by UALCAN (http://ualcan.path.uab.edu/analysis.html) [25]. Immunohistochemistry (IHC) images from The Human Protein Atlas database (https://www.proteinatlas.org/) were used to compare protein expression between ccRCC and normal renal tissues [26].

### 2.2 Survival Analysis of RUNX1/2/3 and Correlations Between RUNX1/2/3 and Clinicopathological Characteristics

Survival data of 527 ccRCC patients with complete survival information were extracted from the TCGA data portal via the “TCGAbiolinks” package. Survival curves for overall survival (OS) were generated by the “survival” package and analyzed by the Kaplan–Meier method and log‐rank tests. The prognostic value of RUNX gene expression on disease‐free survival in ccRCC patients was explored using Gene Expression Profiling and Interactive Analysis (GEPIA, http://gepia.cancer-pku.cn/) [27]. Subsequently, the relationship between RUNX gene expression level and clinicopathological characteristics such as age, gender, tumor grade, tumor TNM stage, depth of tumor infiltration, lymph node metastasis, distant metastasis, and vital status of ccRCC patients was assessed by analyzing the clinical data of ccRCC data sets in the TCGA database. Finally, multivariate Cox regression models were constructed by integrating survival status, several clinicopathological parameters, and gene expression data to assess the prognostic significance of genes, and *p* < 0.05 was considered statistically significant. ROC analysis was performed at 1‐, 3‐, and 5‐year time points using the ROC function of pROC, and AUC values and confidence intervals (CIs) were assessed.

### 2.3 Analyses of Genes Coexpressed With RUNX1/2

Using the cBioPortal online database, the top five coexpressed genes of RUNX1/2 were screened separately according to the adjusted *p* values, and the survival curves of each gene in ccRCC patients were plotted using the GEPIA online tool [28]. Subsequently, 339 RUNX1 coexpressed genes and 91 RUNX2 coexpressed genes were screened (*R* > 0.5, *p* < 0.05), and these two coexpressed gene sets were intersected. Enrichment analysis was performed using the “clusterProfiler” package (version 3.14.3) [29]. *p* < 0.05 and FDR < 0.05 were considered significant enrichment criteria. Gene Ontology (GO) enrichment analysis was performed using GO annotations from the org.Hs.eg.db package (version 3.1.0), and Kyoto Encyclopedia of Genes and Genomes (KEGG) enrichment analysis was performed using the latest KEGG pathway annotations from https://www.kegg.jp/kegg/rest/keggapi.html.

### 2.4 Gene Set Enrichment Analysis of RUNX1/2

Based on the median expression level of RUNX1, we divided the TCGA gene expression data into two groups and used the “edgeR” package for differential expression analysis (FDR < 0.05, |logFC | > 1) [30]. Subsequently, the differentially expressed genes (DEGs) were analyzed using the “clusterProfiler” package to perform Gene Set Enrichment Analysis (GSEA) for GO and KEGG. The hallmark gene set was extracted from MSigDB v7.1 (https://www.gsea-msigdb.org/gsea/msigdb/). The enrichment analysis of RUNX2 was performed by the same method.

### 2.5 Tumor Immune Infiltration Analysis

The “estimate” package was used to score the immune infiltration of tumor tissue. The “MCPCounter” algorithm and the “CIBERSORT” algorithm were used to assess the correlation of RUNX1 and RUNX2 with the presence of various immune cell types and to further quantify the proportion of immune cells in ccRCC samples with high and low expression of RUNX1 and RUNX2 [31, 32].

### 2.6 Analysis of Immune Checkpoint Correlation and Immunotherapy Response

Based on the TCGA gene expression data set, Spearman correlation analysis was used to calculate the correlation between RUNX1 levels and expression of 19 common immune checkpoint genes. Subsequently, all samples were divided into two groups according to the median expression value of RUNX1, and the difference in the expression levels of immune checkpoint genes between the two groups was assessed by the Wilcoxon rank sum test. For immunotherapy response analysis, the “IMvigor210CoreBiologies” package was used to obtain clinical data on immunotherapy response [33]. The effect of RUNX1 expression on the degree of response to immunotherapy in tumor patients was verified by comparing RUNX1 expression between the complete remission/partial remission (CR/PR) group and the stable disease/disease progression (SD/PD) group. Analysis of the correlations between RUNX2 expression and immune checkpoint gene expression and response to immunotherapy was performed in the same manner.

### 2.7 Construction and Evaluation of the RUNX1‐Based Prognostic Nomogram

Based on the multi‐COX regression results, RUNX1 expression and several clinicopathological characteristics (age, gender, stage, and grade) were integrated to build a prognostic model via the “rms” package, and the accuracy of the nomogram was assessed using calibration plots.

### 2.8 Western Blot Analysis

Total protein was extracted from KIRC tissues, adjacent normal kidney tissues, and cultured cells using RIPA lysis buffer with protease inhibitors (Beyotime, China). After quantification with a BCA protein assay kit (Epizyme, China), equal amounts of protein were separated by SDS‐PAGE and transferred onto PVDF membranes. The membranes were blocked with 5% skim milk for 2 h at room temperature, followed by overnight incubation at 4°C with a primary antibody against RUNX1 (25315‐1‐AP, Proteintech, China) RUNX2 (20700‐1‐AP, Proteintech, China), and RUNX3 (27099‐1‐AP, Proteintech, China). After washing with TBST, the membranes were incubated with an HRP‐conjugated secondary antibody for 1 h at room temperature. Protein signals were detected using an ECL kit (Fdbio Science, China), with GAPDH (Proteintech, China) as a loading control.

### 2.9 RNA Extraction and RT‐qPCR Analysis

Total RNA was extracted using TRIzol (Invitrogen, USA), and cDNA was synthesized with the Hifair II 1st Strand cDNA Synthesis Kit (YEASEN, China). RT‐qPCR was performed using Hieff qPCR SYBR Green Master Mix (YEASEN, China). The cycling conditions included an initial denaturation at 95°C for 5 min, followed by 40 cycles of 95°C for 10 s, 55°C for 20 s, and 72°C for 30 s. GAPDH served as an internal control, and gene expression was analyzed using the 2^−ΔΔCt method. The specific primer sequences used were as follows: GAPDH (forward: ACACCCACTCC TCCACCTTTG, reverse: TCCACCACCCTGTTGCTGTAG), RUNX1 (forward: AGACCCTGCCCATCGCTTTC, reverse: TTCATCATTGCCAGCCATCACAG), RUNX2 (forward: CCACCACTCACTACCACACCTAC, reverse: TCCTGACGAA GTGCCATAGTAGAG), and RUNX3 (forward: ACCAGCCGCTTCCACCATAC, reverse: GCCAGAGGATGTCCCGTAGTAG).

### 2.10 Statistical Analysis

The statistical analysis and construction of graphics for this study were mainly performed using R programming language using the “TCGAbiolinks” package, the “clusterProfiler” package, the “survival” package, the “rms” package, and the “ggplot2” package, etc. For statistical analysis, nonparametric or parametric tests were selected according to the type of data. The Wilcoxon rank sum test was used for comparison of two groups of data, and the Kruskal–Wallis test was used for comparison of multiple groups of data. The Benjamini–Hochberg method was chosen for differential gene expression analysis and enrichment analysis to correct *p* values and convert to FDR as screening criteria. The Kaplan–Meier method was used to depict survival analysis, and significance was statistically tested by log‐rank test. Multivariate analyses were performed using the Cox proportional hazards regression model. *p* < 0.05 was considered statistically significant. *p < 0.05, **p < 0.01, ***p < 0.001.

## 3 Results

### 3.1 Transcription Level of RUNX1/2/3 in ccRCC

First, the transcriptional level of RUNX1/2/3 among multiple human cancers and normal tissues was retrieved using the TIMER database. The results are presented in Figure 1A. Three RUNX genes were significantly highly expressed in breast invasive carcinoma, renal clear cell carcinoma (ccRCC), renal papillary cell carcinoma (KIRP), lung adenocarcinoma, stomach adenocarcinoma (STAD), and thyroid carcinoma (THCA). This result tentatively suggested that RUNX genes may play some critical role in tumor progression. Next, to further investigate the changes in the transcript levels of the RUNX genes in ccRCC, we performed differential analysis of mRNA expression between tumor and normal samples in two GEO data sets (GSE53757 and GSE76351) and the TCGA data set using the “limma” package. The expression of DEGs from the above three data sets was presented in volcano plots and intersected with the transcription factor data set using Venn diagram analysis (Figure 1B,C). The results showed that a total of 48 transcription factors showed significant differential expression in all three data sets. In addition, the RUNX transcription factor family was the only gene family that was significantly overexpressed across the conditions tested. Moreover, we analyzed the significance of differences in the mRNA expression levels of RUNX1/2/3 in ccRCC and normal renal tissues in the three data sets using the Wilcoxon rank sum test. The results showed that the RUNX family was highly expressed in tumor tissues, and the differences were statistically significant (Figure 1D). Western blot analysis demonstrated that RUNX1, RUNX2, and RUNX3 protein levels were significantly upregulated in ccRCC cell lines compared to normal renal tubular epithelial cells (HK‐2) (Figure 2A–D). Quantitative analysis confirmed this increase, suggesting a potential role for RUNX family members in ccRCC progression. Consistently, RT‐qPCR results showed significantly higher mRNA expression of RUNX1, RUNX2, and RUNX3 in ccRCC cells than in normal kidney cells (Figure 2E–G). Moreover, analysis of clinical samples revealed elevated RUNX1, RUNX2, and RUNX3 protein expression in ccRCC tissues compared to adjacent normal tissues (Figure 2H). These findings highlight the potential involvement of RUNX family members in ccRCC pathogenesis and their relevance as molecular markers or therapeutic targets. In addition, the association of the levels of all RUNX genes with ccRCC subtypes was analyzed by retrieving data from the UALCAN database. The results showed that RUNX1 and RUNX2 levels were significantly correlated with different subtypes, while RUNX3 levels were not (Figure S1A–C).

**Figure 1:**
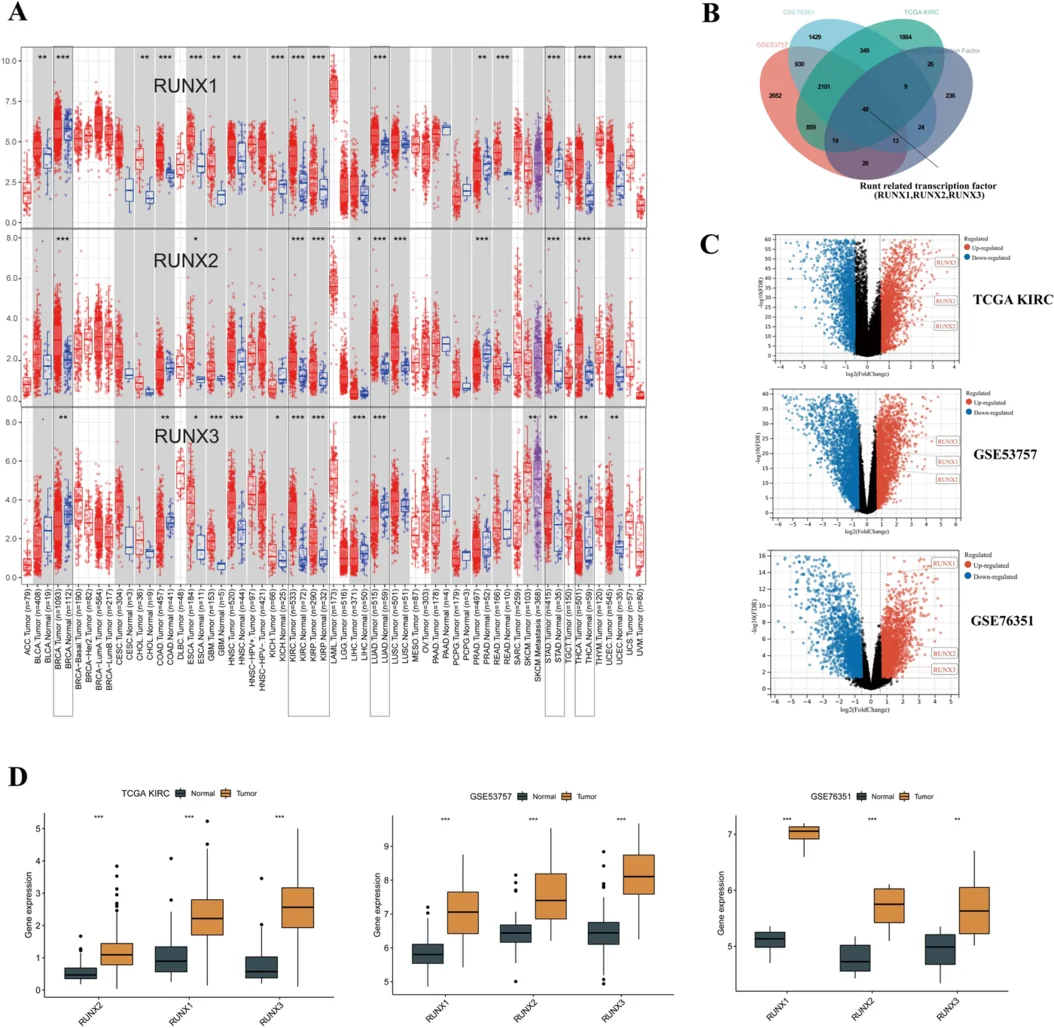
The expression levels of RUNX genes are upregulated in renal clear cell carcinoma (ccRCC). (A) The expression levels of RUNX1, RUNX2, and RUNX3 expression in multiple human cancers and normal tissues were analyzed in TIMER. (B) Venn diagram analysis of differentially expressed transcription factors in TCGA, GSE53757, and GSE76351 data sets. (C) Volcano plot of the distribution of all differentially expressed genes in the TCGA, GSE53757, and GSE76351 data sets. (D) Differential expression of RUNX1, RUNX2, and RUNX3 in ccRCC tissues and normal tissues based on TCGA, GSE53757, and GSE76351 data sets. *p < 0.05, **p < 0.01, ***p < 0.001, ****p < 0.0001.

**Figure 2:**
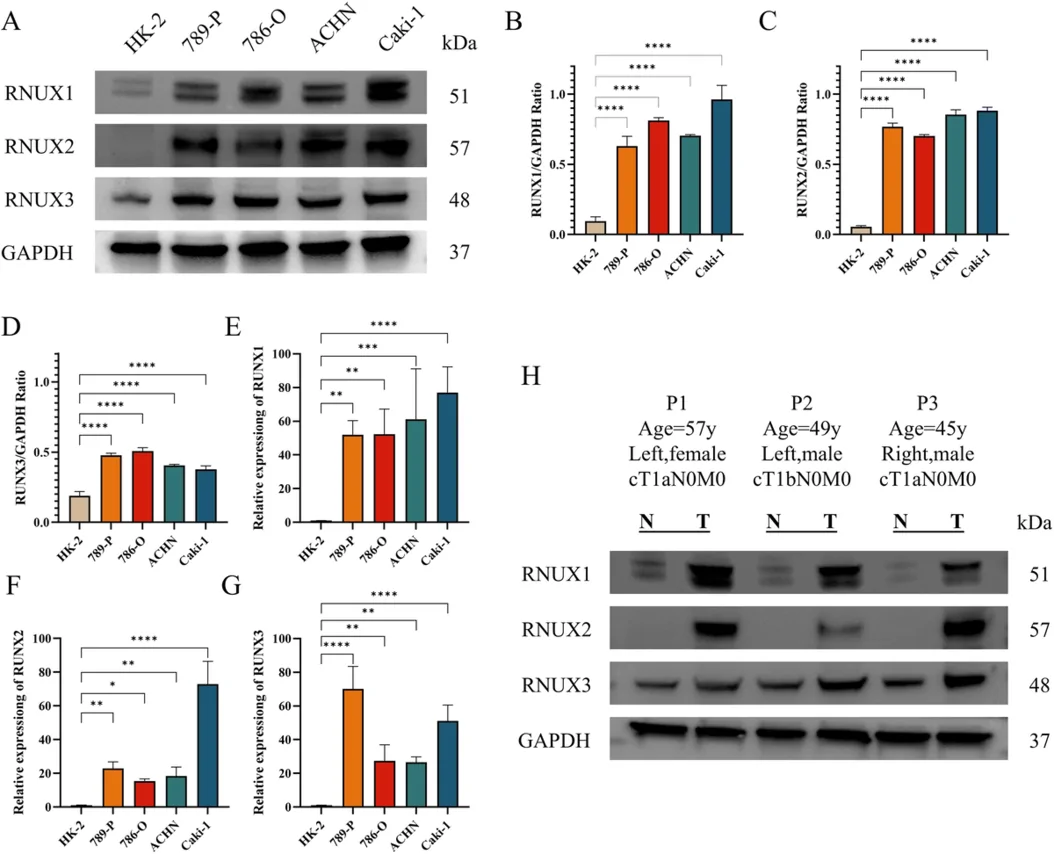
Expression verification on RUNX1, RUNX2, and RUNX3 in ccRCC. (A) RUNX1, RUNX2, and RUNX3 protein‐level expression in normal kidney and ccRCC cells. (B–D) Quantitative analysis of RUNX1, RUNX2, and RUNX3 protein expression levels based on relative band intensity. (E–G) RUNX1, RUNX2, and RUNX3 mRNA‐level expression in normal kidney and ccRCC cells. (H) RUNX1, RUNX2, and RUNX3 protein‐level expression in clinical ccRCC and adjacent normal tissues. L, lefe kidney, N, normal control; R, right kidney; T, ccRCC tissue.

### 3.2 The Prognostic Value of RUNX1/2/3 Genes in ccRCC Patients

Genes that are highly expressed in tumor tissues may be strongly associated with tumor prognosis. Therefore, we explored the use of highly expressed RUNX genes in the prognosis of ccRCC patients. The 527 ccRCC patients in the TCGA database were divided into RUNX1 high and low expression groups according to the median mRNA values of RUNX1 levels, and survival curves were plotted by Kaplan–Meier and evaluated by log‐rank test. Subsequently, the same treatment and analysis were performed according to the median expression values of RUNX2 and RUNX3 levels. As shown in Figure 3A–C, higher RUNX1 (HR = 2.15, 95% CI: 1.57–2.94, and *p* < 0.001) and RUNX2 (HR = 1.58, 95% CI: 1.17–2.15, and *p* = 0.003) expression was significantly related to unfavorable OS, while RUNX3 expression (HR = 1.16, 95% CI: 0.86–1.57, and *p* = 0.33) was not. Next, the results of ROC analysis showed that RUNX1 and RUNX2 had good prediction accuracy for ccRCC patients, and the areas under the ROC curve for discrimination of 1‐year survival were 0.67 and 0.62, respectively (Figure 3D–F). Furthermore, we analyzed the use of RUNX gene expression in the prediction of the disease‐free survival (DFS) of ccRCC patients through the GEPIA online database (Figure S2A–C). In contrast, we found that only RUNX1 expression (HR = 1.90, *p* < 0.001) had a significant correlation with disease‐free survival, and ccRCC patients with higher transcriptional levels of RUNX1 had a shorter DFS.

**Figure 3:**
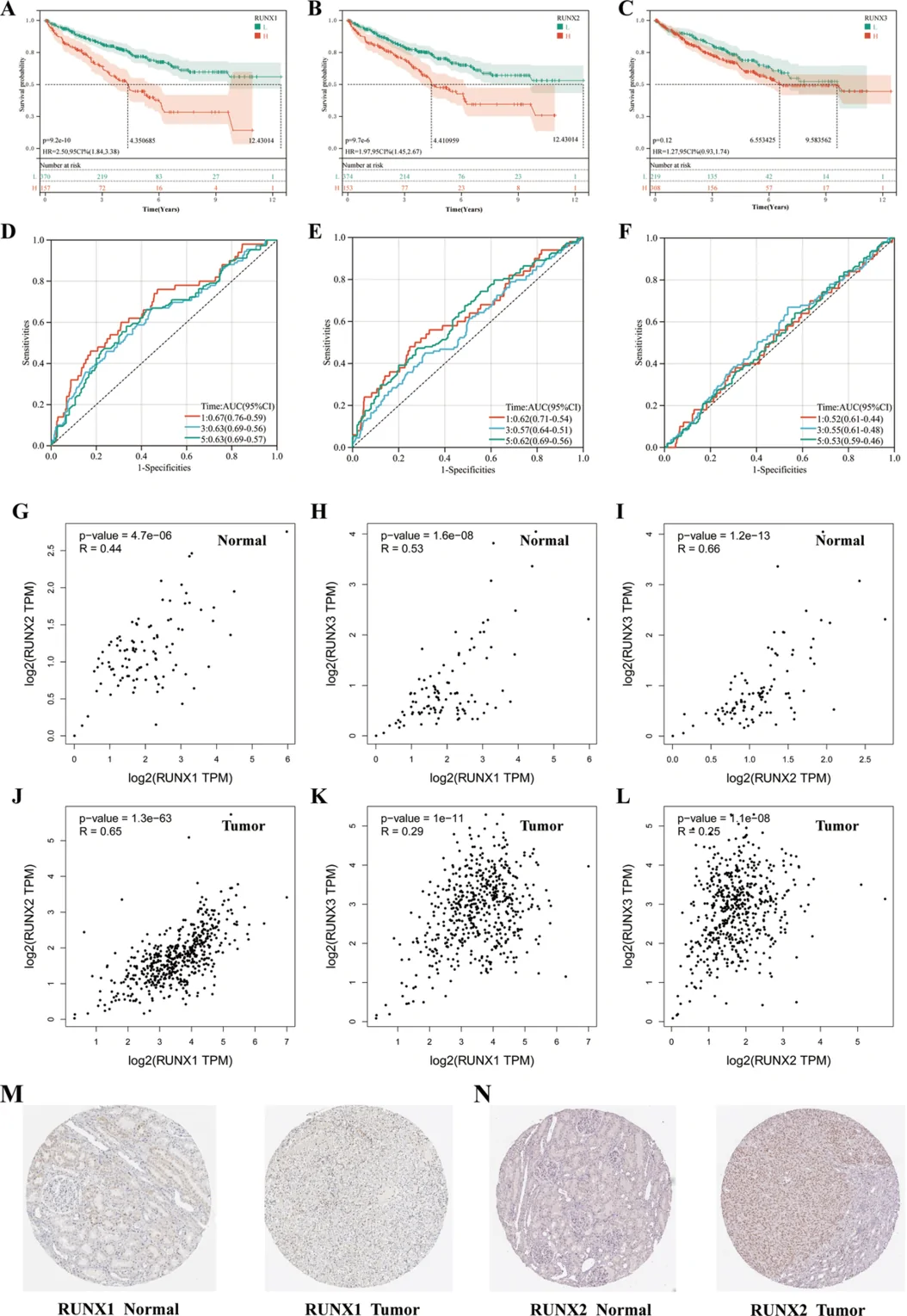
Prognostic impact of RUNX1, RUNX2, and RUNX3 on ccRCC patients and correlation analysis between the three genes. (A–C) Kaplan–Meier plots comparing high or low expression of RUNX1/2/3 on OS using the log‐rank test. (D–F) Time‐dependent ROC curves of RUNX1/2/3 expression on OS in ccRCC patients. (G–L) Comparison of correlated changes of RUNX1/2/3 in normal tissues and ccRCC tissues. (M, N) Protein expression of RUNX1 and RUNX2 in ccRCC tissues and normal tissues.

Given that the three genes are of the same family and are all highly expressed in ccRCC, there may be significant coexpression. Therefore, we analyzed the correlation between RUNX1, RUNX2, and RUNX3 levels in ccRCC tumor tissue and normal renal tissue. We found that only the correlation between RUNX1 and RUNX2 expression was significantly higher in tumor tissues than in normal tissues, while correlations of RUNX1 and RUNX3 levels, as well as RUNX2 and RUNX3 levels, were significantly lower in tumor tissues than in normal tissues (Figure 3G–L). The correlation results combined with the above analysis of gene expression and prognostic value suggest that there is a cluster regulation of RUNX1 and RUNX2 in ccRCC affecting ccRCC progression and patient prognosis. In addition, we compared the protein levels of RUNX1 and RUNX2 by IHC staining obtained from the Human Protein Atlas database. As shown in Figure 2M,N, the protein expression level of RUNX1/2 was higher in ccRCC than in normal renal tissue.

### 3.3 Relationship Between RUNX1/2 and Clinicopathological Characteristics of ccRCC Patients

Using the clinical information of ccRCC patients with samples in the TCGA database, we explored the relationship between the mRNA expression of RUNX1/2 and the clinicopathological characteristics of ccRCC patients (Figure 4). The results revealed that the mRNA expression of RUNX1 was significantly increased in males (*p* < 0.01), the G3/4 phase (*p* < 0.05), advanced stages (III/IV) (*p* < 0.001), advanced tumor status (T3/4, N1 and M1) (*p* < 0.001, *p* < 0.01 and *p* < 0.01), and death status (*p* < 0.001), while the mRNA expression of RUNX2 was also significantly higher in males (*p* < 0.05), the G3/4 phase (*p* < 0.05), advanced stages (III/IV) (*p* < 0.001), advanced tumor status (T3/4 and M1) (*p* < 0.01 and *p* < 0.01), and death status (*p* < 0.001). These results suggest that RUNX1 and RUNX2 may function synergistically as coexpressed genes in ccRCC.

**Figure 4:**
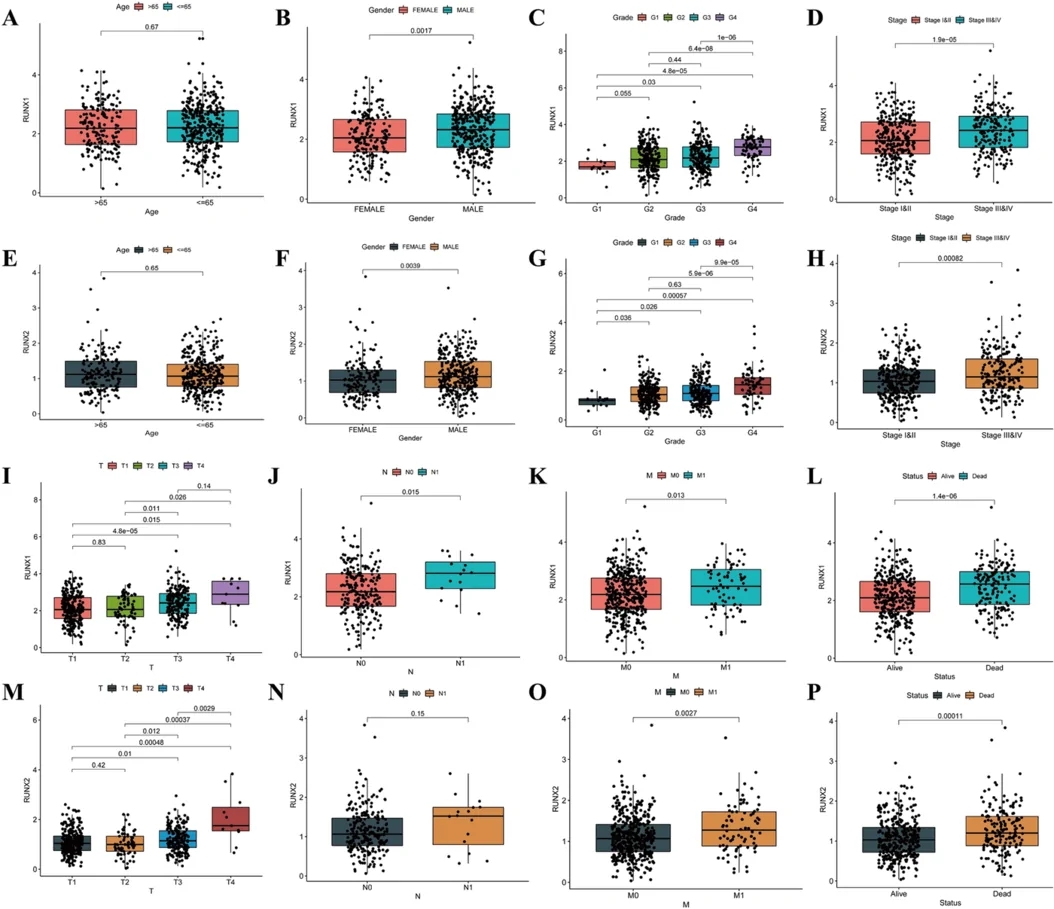
Association between RUNX1/2 expression and clinicopathological characteristics in ccRCC patients. The mRNA expression levels of RUNX1/2 are shown with scatter plots using the TCGA‐KIRC data set according to age (A, E), gender (B, F), grade (C, G), stage (D, H), tumor stage (I, M), lymph node status (J, N), metastasis stage (K, O), and status (L, P).

### 3.4 Analysis of the RUNX1/2 Coexpression Gene Network Indicates Their Collective Involvement in Immunoregulatory Processes in the Tumor Microenvironment

As shown in Figure 5A,B, the top five coexpressed genes of RUNX1 or RUNX2, which are TGFBI, ADAM12, ETV6, GALNT2, C1R, AXL, and IFI16, were identified through the cBioPortal online database. OS survival curves of these genes in ccRCC were plotted by KM‐Ploter, and the results showed that all coexpressed genes except AXL significantly affected prognosis, indicating that these coexpressed genes were significantly associated with poor prognosis in ccRCC (Figure 5C).

**Figure 5:**
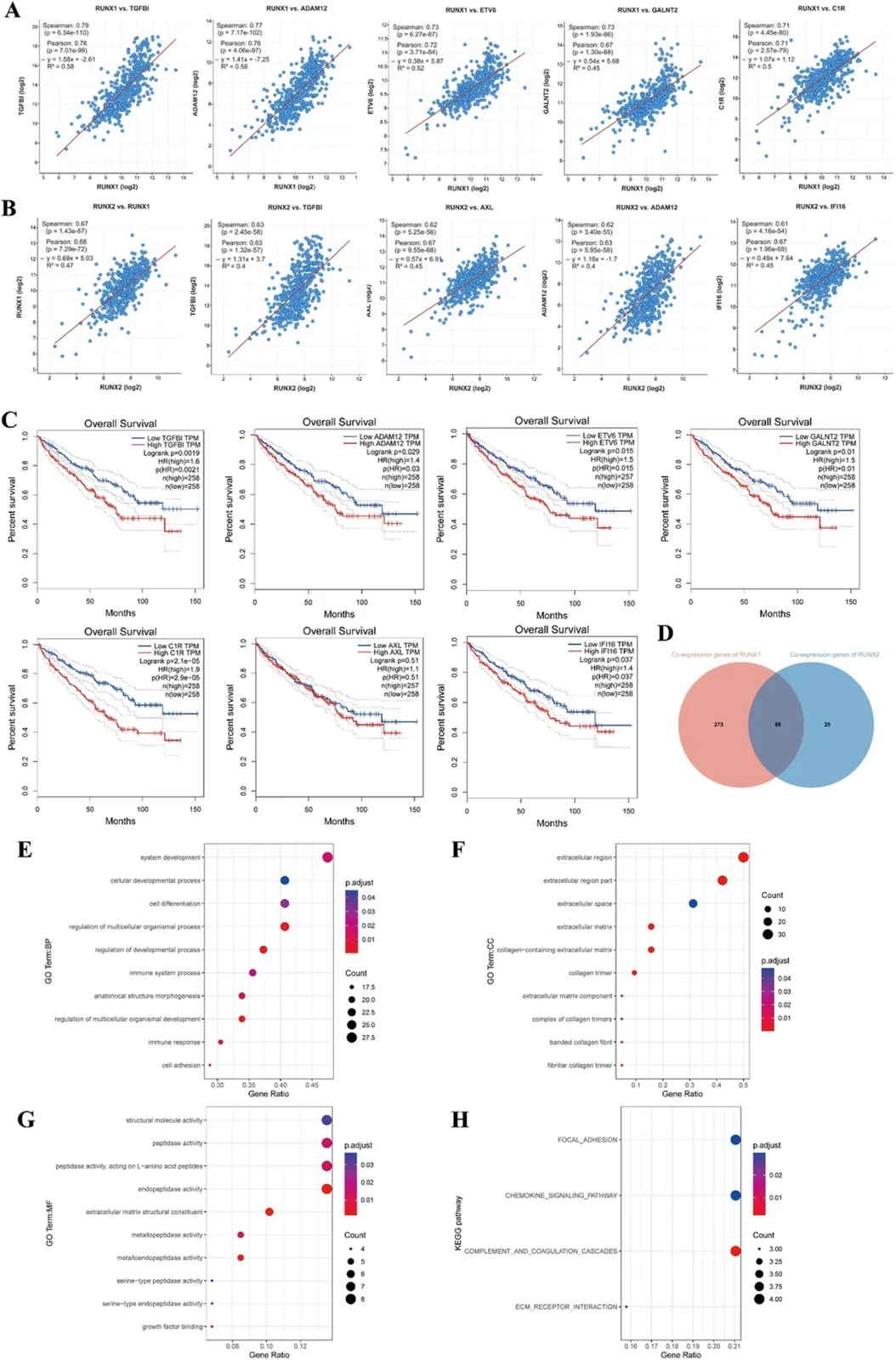
Prognostic and functional analysis of co‐expressed genes of RUNX1 and RUNX2. (A, B) The top five co‐expressed genes of RUNX1 and RUNX2 were screened separately using the cBioPortal online database, and correlation analysis was performed. (C) The impact of expression levels of seven co‐expressed genes on the OS of ccRCC patients was evaluated using the GEPIA online tool. (D) Co‐expressed genes of RUNX1 and RUNX2 (*r* > 0.5) were screened separately using the cBioportal database and subjected to Venn diagram analysis, in which 66 genes were significantly co‐expressed with both RUNX1 and RUNX2. (E–H) GO and KEGG enrichment analysis of the co‐expressed gene network obtained in (D) to explore the potential biological functions.

Subsequently, we filtered out 339 RUNX1 coexpressed genes and 91 RUNX2 coexpressed genes using a correlation coefficient of 0.5 as the cutoff value. To explore the potential biological functions shared by RUNX1 and RUNX2 in the development of ccRCC, we intersected the above genes and constructed coexpression gene networks for GO and KEGG enrichment analyses (Figure 5D–H). Among the top 10 enriched functions in the biological process (BP) category, system development, cell differentiation, immune system process, immune response, and cell adhesion are associated with the tumorigenesis and progression of ccRCC. In the top four KEGG‐enriched entries, focal adhesion, chemokine signaling pathway, complement and coagulation cascades, and extracellular matrix receptor interaction were revealed, and all are closely associated with tumorigenesis and progression. Among them, the chemokine signaling pathway and complement and coagulation cascades are involved in immune regulation, while focal adhesion and extracellular matrix receptor interactions are closely related to the regulation of the tumor immune microenvironment. The above enrichment analysis results suggest that RUNX1 and RUNX2 may be involved in the immune regulation of the tumor microenvironment during the development and progression ofccRCC.

### 3.5. GSEA Identified That RUNX1/2 Participated in Several Immune‐Related Pathways in ccRCC

3.5

To further analyze the possible biological pathways of RUNX1 and RUNX2 in ccRCC, we screened the DEGs based on the median value of RUNX1 or RUNX2 expression and performed GSEA for GO and KEGG. Notably, the results of GO analysis for RUNX1 and RUNX2 were remarkably similar. As shown in Figure 6A and D, the enrichment results of the BP category indicated that RUNX1 and RUNX2 participated in the immune response and inflammatory processes (RUNX1: regulation of leukocyte activation, regulation of lymphocyte activation, T‐cell activation, adaptive immune response, positive regulation of cytokine, regulation of hemopoiesis, regulation of cell–cell adhesion, positive regulation of cell adhesion; RUNX2: regulation of leukocyte activation, positive regulation of cell adhesion, regulation of hemopoiesis, T‐cell activation, positive regulation of cytokine production, positive regulation of secretion by cell, positive regulation of defense response and regulation of innate immune response). In the molecular function category, growth factor receptor binding, growth factor activity, cytokine activity, cytokine receptor binding, receptor–ligand activity, and G protein‐coupled receptor binding were mainly enriched (Figure S3A,B). KEGG enrichment analysis also showed similar results (Figure 6C,D). The top three KEGG pathways of both RUNX1 and RUNX2 were cytokine–cytokine receptor interaction, chemokine signaling pathway, and JAK‐STAT signaling pathway, which are involved in immune response and inflammatory processes. In addition, other pathways with high enrichment, such as transcriptional misregulation in cancer, microRNAs in cancer, the PI3K‐AKT signaling pathway, focal adhesion, and the Wnt signaling pathway, are also widely involved in cell adhesion and tumorigenesis.

**Figure 6:**
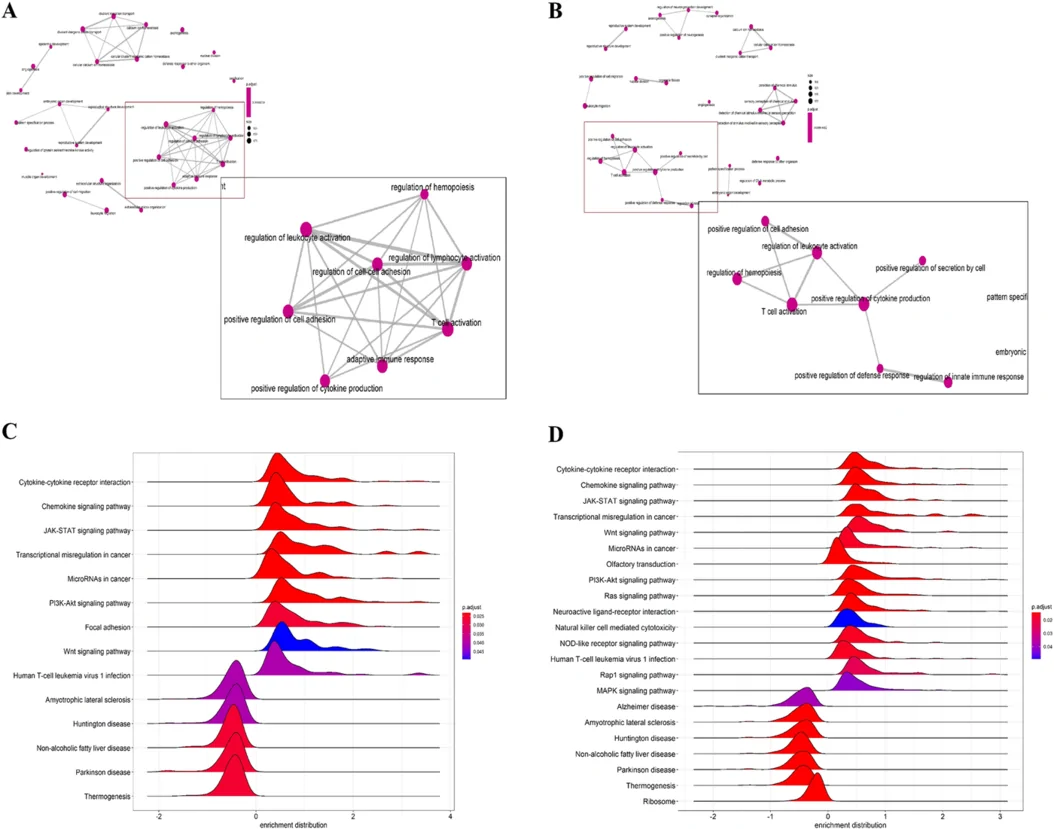
Enrichment analysis of differentially expressed genes based on RUNX1/2 high and low expression ccRCC tissues. (A, C) GO(BP) and KEGG enrichment analysis of differentially expressed genes between high and low RUNX1 mRNA expression groups based on gseGO. (B, D) GO(BP) and KEGG enrichment analysis of differentially expressed genes between high and low RUNX2 mRNA expression groups based on gseKEGG.

### 3.6 RUNX1/2 Promotes Immunosuppressive Microenvironment Formation in ccRCC

As shown in Figure 7A, immune infiltration score analysis indicated that ccRCC tissues with high expression of RUNX1 and RUNX2 received higher scores. Therefore, the “MCPCounter” algorithm was used to assess the correlation between the expression of RUNX1 or RUNX2 in ccRCC samples and the infiltration of 10 immune cell types in the tumor microenvironment. We found that RUNX1 and RUNX2 levels were significantly correlated with levels of each type of immune cell in the tumor microenvironment (Figure 7B). RUNX1 expression was strongly positively correlated with the presence of fibroblasts (*r* = 0.597, *p* < 0.001), followed by B lineage (*r* = 0.351, *p* < 0.001), monocytic lineage (*r* = 0.245, *p* < 0.001), and T cells (*r* = 0.220, *p* < 0.001); RUNX2 expression also had a strong positive correlation with the presence of fibroblasts (*r* = 0.444, *p* < 0.001) and a moderate correlation with the presence of many other immune cell types except neutrophils. Moreover, we divided ccRCC patients into two groups based on the high or low expression of RUNX1 or RUNX2 mRNA and compared the differences in the infiltration of several types of immune cells that were more strongly correlated with either RUNX1 or RUNX2 in the two groups. The results showed that the infiltration scores of all these types of immune cells were significantly different between the two groups (Figure 7C,E).

**Figure 7:**
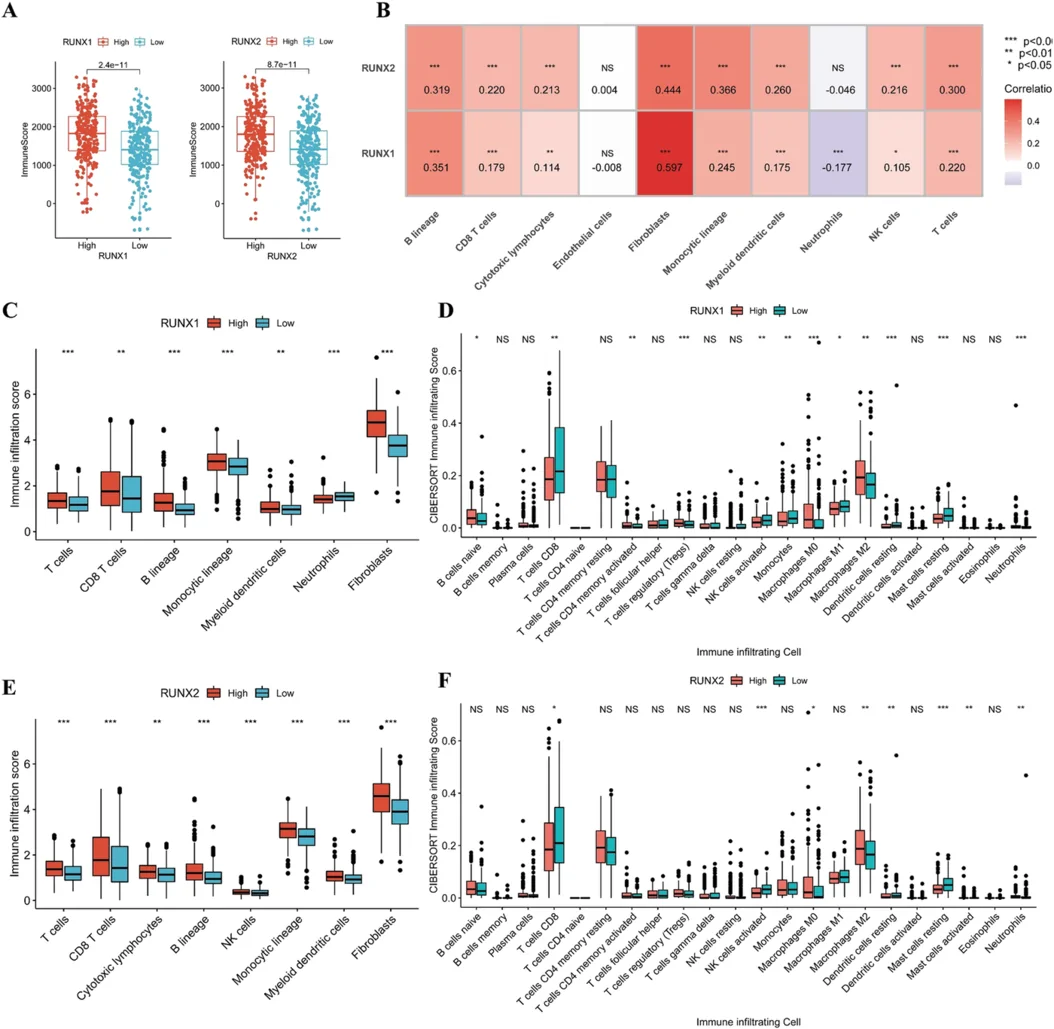
Correlation analysis between RUNX1 and RUNX expression and various types of immune cell infiltration in ccRCC tissues. (A) The influence of RUNX1/2 expression in ccRCC on immune infiltration scores. (B) The MCPCounter algorithm calculated the correlation between RUNX1/2 expression and infiltration of 10 immune cells in ccRCC. (C, E) Differences in the degree of immune cell infiltration between high and low RUNX1/2 expression groups. (D, F) CIBERSORT algorithm calculates the difference in infiltration of 22 immune cell subtypes between RUNX1/2 high and low expression groups. *p < 0.05, **p < 0.01, ***p < 0.001.

To further clarify the role of RUNX1 and RUNX2 in the tumor microenvironment, we evaluated the influence of RUNX1 or RUNX2 on tumor infiltration by 22 immune cells, including multiple cell subtypes, using the “CIBERSORT” algorithm. We found that CD8+ T cells, activated NK cells, M1 macrophages, monocytes, dendritic cells, and mast cells exhibited significantly less infiltration of tumor tissues with high RUNX1 expression, while regulatory T cells (Tregs), M0/M2 macrophages, and neutrophils exhibited significantly more infiltration (Figure 7D). Relatively, in tumor tissues with high RUNX2 expression, CD8+ T cells, NK cells, dendritic cells, and mast cells infiltrated less, and only M0/M2 macrophages and neutrophils infiltrated more (Figure 7F). To further verify the above results, TISIDB database was used to evaluate the correlation between several immune cells and the expression of RUNX1 and RUNX2 (Table 1). The above results indicated that the RUNX1 and RUNX2 expression were strongly correlated with macrophages, myeloid‐derived suppressor cells, Th1 cells, Th2 cells, Tregs, and Tfh cells, and moderately correlated with active B cells, but not with neutrophils.

**Table 1:** Correlation analysis of RUNX1 and RUNX2 with some immune cells in TISIDB.

	KIRC RUNX1		KIRC RUNX2	
Immune cells	rho	*p*	rho	*p*
Active B cells	0.282	< 0.001	0.339	< 0.001
Macrophages	0.550	< 0.001	0.477	< 0.001
MDSCs	0.442	< 0.001	0.451	< 0.001
Th1 cells	0.460	< 0.001	0.447	< 0.001
Th2 cells	0.505	< 0.001	0.431	< 0.001
Tregs	0.468	< 0.001	0.445	< 0.001
Tfh cells	0.410	< 0.001	0.397	< 0.001
Neutrophils	0.016	0.713	0.082	0.057

*Note: p* < 0.05, statistically significant; rho and *p* value of Spearman correlation test.

Abbreviations: ccRCC, clear cell renal cell carcinoma; MDSCs, myeloid‐derived suppressor cells; RUNX, runt‐related transcription factors; Tfh, follicular helper T cells; Th, T helper cells; TISIDB, an integrated repository portal for tumor‐immune system interactions; Tregs, regulatory T cells.

### 3.7 Immunotherapy Response

Immune checkpoints are a class of immunosuppressive molecules that play an important role in the formation of the tumor immunosuppressive microenvironment and tumor immunotherapy. Therefore, we calculated the correlation of RUNX1/2 expression in ccRCC with that of 19 common immunotherapy checkpoint genes. As shown in Figure 8A–C, we found that the expression of RUNX1 in ccRCC showed a significant positive correlation with expression of BTLA, CD96, CSF1R, CTLA4, LAG3, LGALS9, PDCD1LG2, TGFB1 and TIGIT, while RUNX2 expression showed a significant positive correlation with expression of BTLA, CD160, D244, CD96, CSF1R, CTLA4, LAG3, LGALS9, PDCD1, PDCD1LG2, TGFB1 and TIGIT. Next, the effect of RUNX1 and RUNX2 expression on immunotherapy response was evaluated visually by comparing the differences in RUNX1 and RUNX2 expression between the CR/PR and SD/PD groups in the IMvigor210 cohort. We found that RUNX1 expression was significantly negatively correlated with the efficacy of anti‐PDL treatment in tumor patients, while RUNX2 expression was not (Figure 8D,F). In addition, Kaplan–Meier survival analysis was used to evaluate the impact of RUNX1 and RUNX2 expression on patient prognosis in the immunotherapy response cohort which showed that the group with high RUNX1 expression had a worse prognosis, while RUNX2 expression was not statistically associated with patient prognosis (Figure 8E,G). We explored the effect of RUNX1 and RUNX2 on the expression of several immune checkpoints (PDCD1, CTLA‐4, LAG3, TIGIT, and BTLA) that have been shown to affect prognostic survival in ccRCC based on GEPIA online analysis. we found significant correlations of RUNX1 and RUNX2 with PDCD1 (rho = 0.206, *p* < 0.001; rho = 0.257, *p* < 0.001), CTLA‐4 (rho = 0.244, *p* < 0.001; rho = 0.357, *p* < 0.001), LAG3 (rho = 0.249, *p* < 0.001; rho = 0.271, *p* < 0.001), TIGIT (rho = 0.298, *p* < 0.001; rho = 0.344, *p* < 0.001) and BTLA (rho = 0.302, *p* < 0.001; rho = 0.415, *p* < 0.001) in Figure S4A–J. The above results suggest that RUNX1 may hamper anti‐PD‐L1 immunotherapy by modulating multiple immune checkpoints.

**Figure 8:**
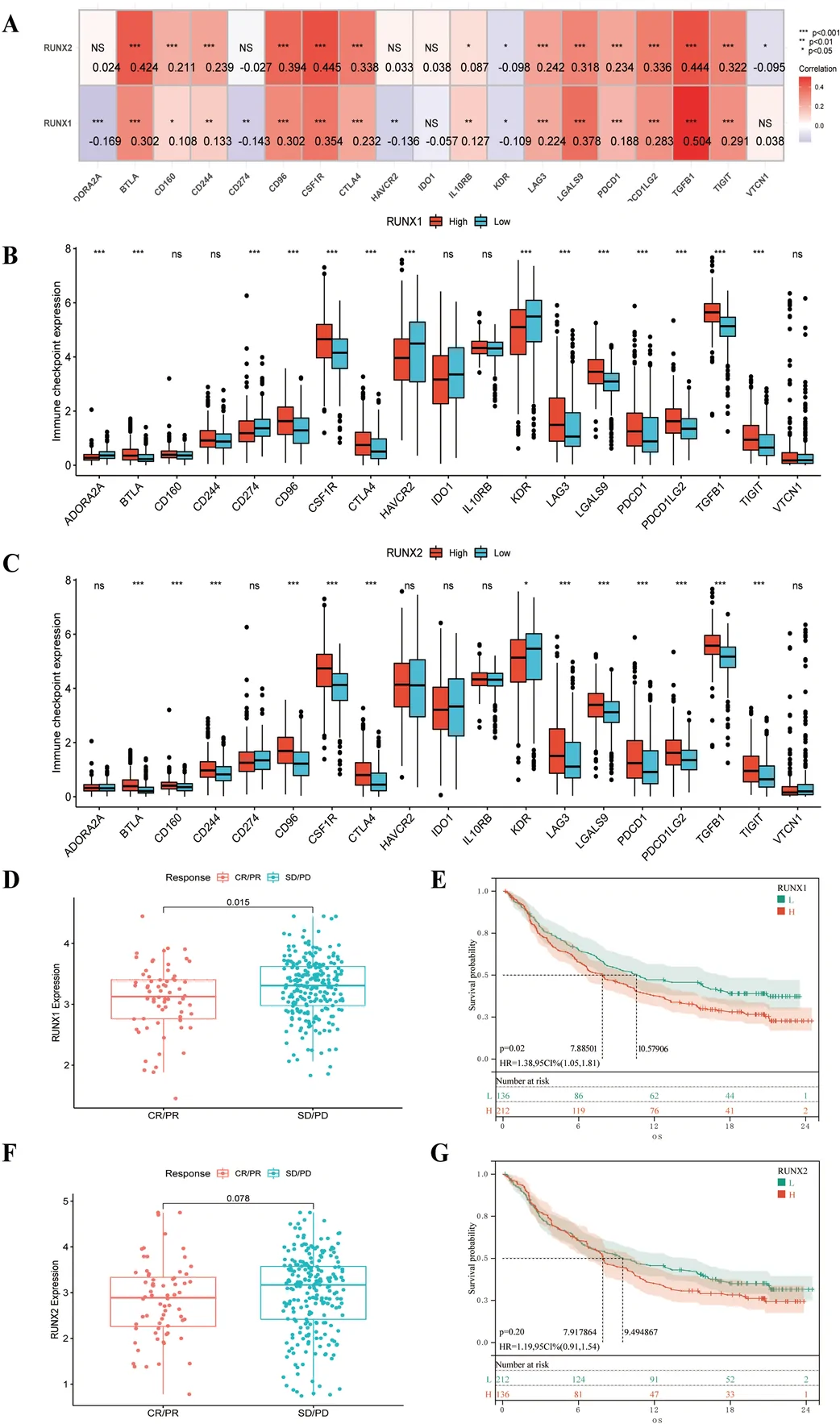
Analysis of the effect of RUNX1/2 expression on immunotherapy in ccRCC patients. (A) Correlation between RUNX1/2 expression and multiple immune checkpoints in ccRCC tissues based on TCGA database. (B, C) Differences in expression of multiple immune checkpoints between high and low RUNX1/2 expression groups. (D–G) Investigating the effect of RUNX1/2 expression on the degree of response to immunotherapy in oncology patients and the impact on patient prognosis in the IMvigor210 cohort. *p < 0.05, **p < 0.01, ***p < 0.001.

### 3.8 Development and Assessment of the RUNX1‐Based Prognostic Model

To further clarify whether RUNX1 and RUNX2 expression can be used as independent prognostic biomarkers, we performed multifactorial Cox regression analysis using the “survival” package based on 494 ccRCC samples from the TCGA database. The results suggest that RUNX1 expression can be used as an independent prognostic biomarker for ccRCC (for OS, HR = 1.66, 95% CI = 1.35–2.05, *p* < 0.001), while RUNX2 expression cannot (Figure 9A). To better use RUNX1 expression in clinical practice, we further integrated four clinicopathological parameters of ccRCC patients in the TCGA database, including age, gender, stage, grade, and RUNX1 expression, and created a nomogram based on the Cox method to predict OS at 1, 3, and 5 years. The results showed that the overall C‐index of the model was 0.775, 95% CI = 0.741–0.809, *p* < 0.001 (Figure 9B,C). The calibration curves for 1‐year, 3‐year, and 5‐year OS showed that the nomogram‐predicted OS closely matched the actual survival of ccRCC patients. Subsequently, we used ROC curves to assess the accuracy of the nomogram for predicting ccRCC prognosis and showed that the AUC values for predicting 1‐year, 3‐year, and 5‐year survival were 0.87, 0.81, and 0.78, respectively, demonstrating very high accuracy (Figure 9D). Finally, we performed ROC analysis of 1‐year survival predicted using various clinical prognostic factors, including age, gender, RUNX1 expression, stage, grade, and RUNX1‐based nomogram, and the results showed that the RUNX1‐based nomogram had the highest value in determining the prognosis of ccRCC patients, with an AUC = 0.872 (Figure 9E).

**Figure 9:**
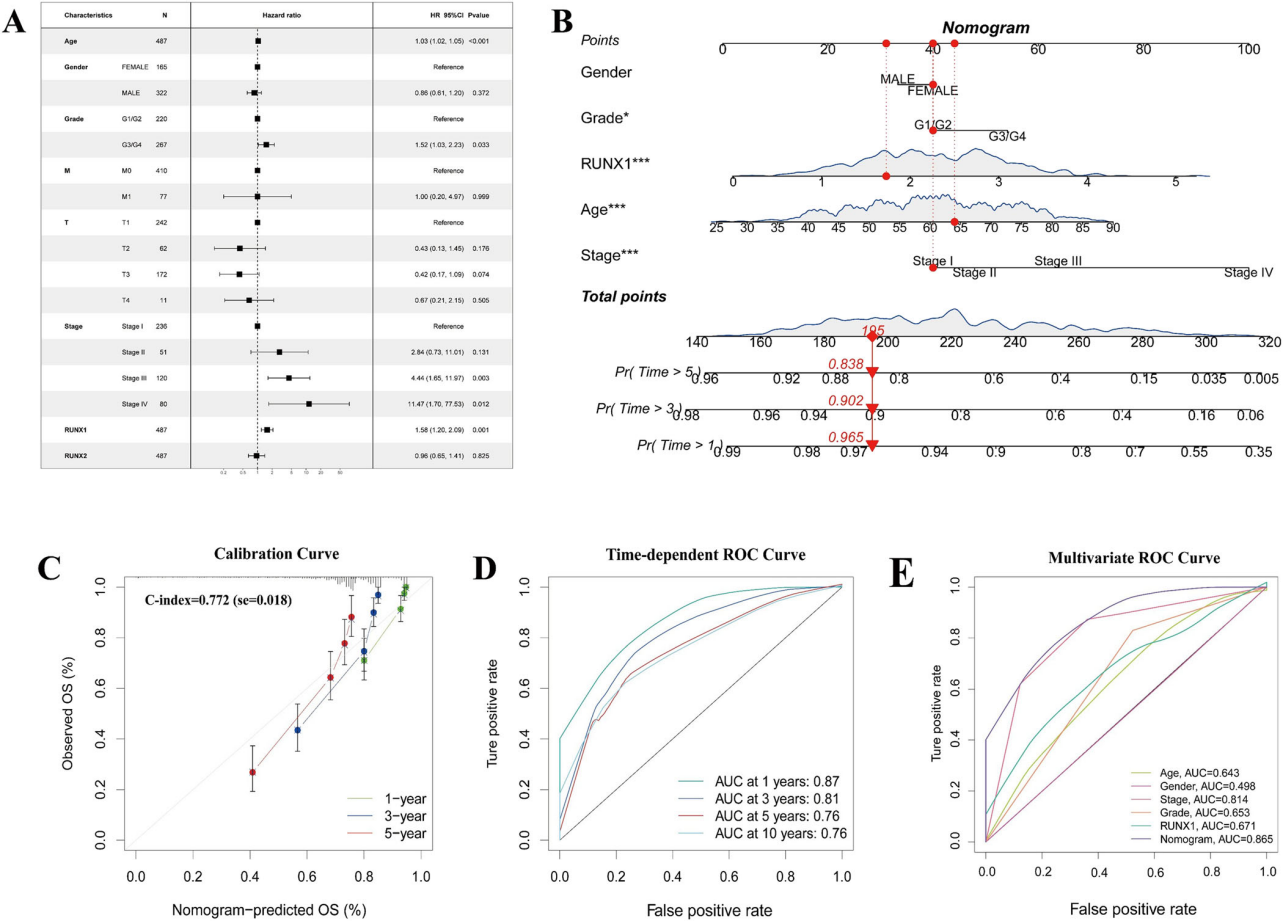
Construction of RUNX1‐based prognostic model for ccRCC. (A) Multivariate COX regression analysis of RUNX1/2 and other prognostic factors in ccRCC patients. (B) A nomogram includes prognostic features such as age, gender, grade, stage, and RUNX1 expression. (C) The calibration chart of nomogram in (B). (D) ROC curve was used to assessed the accuracy of this Nomogram in predicting prognosis. (E) ROC comparison of 1‐year OS for multiple clinical factors. *p < 0.05, **p < 0.01, ***p < 0.001.

## 4 Discussion

RCC is one of the most common urological malignancies worldwide. As the most common subtype, ccRCC is usually asymptomatic in the early stages of disease onset, resulting in patients more often progressing to advanced disease stages with poor cancer‐specific survival rates [34]. ccRCC is considered a tumor with high degrees of immune infiltration, and with the advent of ICIs, the prognosis of metastatic clear cell renal cell carcinoma (mccRCC) has changed dramatically. However, there are still many patients who develop primary or secondary drug resistance due to physical or tumorigenic reasons [4]. Among them, the tumor microenvironment plays an important role in inducing ICI resistance as it is the main interface of the tumor [35]. In addition, genetic alterations have been found to be closely associated with the development and progression of ccRCC and the regulation of the tumor immune microenvironment [36]. Studies from Ueda et al. and Mikami et al. demonstrated that immune infiltration‐related genes (IIRGs) are strongly associated with the immunotherapeutic response and clinical prognosis of ccRCC patients [37, 38]. Therefore, identifying key immune‐related biomarkers and combining these markers with prognosis‐related clinicopathological parameters to construct accurate prognostic models is an effective way to improve the survival time and quality of life for patients and to improve their prognosis.

In recent years, there has been increasing evidence that RUNX genes have crucial roles in tumorigenesis, progression and metastasis, tumor immunosuppression, and other aspects through direct and indirect actions. However, the tumor regulatory mechanism of RUNX genes in ccRCC remains to be explored, and RUNX gene expression and its effect on immune infiltration and immunotherapeutic response in ccRCC have not been studied. Previous studies have shown that RUNX1 expression is significantly elevated in ccRCC and that its expression and methylation levels are significantly correlated with prognosis, whereas RUNX2 and RUNX3 expression in ccRCC and their effects on prognosis are contradictory. In this study, by comprehensively analyzing the TCGA data set and two GEO data sets and performing Cox regression analysis and ROC curve analysis on their survival data, we fully demonstrated that RUNX1, RUNX2, and RUNX3 were all highly expressed in ccRCC. Among them, the expression of RUNX1 and RUNX2 was associated with patient prognosis, and RUNX3 expression was not associated with patient prognosis. In addition, we confirmed that RUNX1 and RUNX2 expression was associated with grade, TNM stage, invasion depth, lymph node metastasis, and vital status in ccRCC patients. These results tentatively suggest that RUNX1 and RUNX2 are oncogenes in ccRCC and have the potential to serve as prognostic biomarkers for patients.

To investigate the cluster regulation of RUNX family members in ccRCC, we performed a comparative analysis of the correlation between RUNX1/2/3 in tumor tissues and normal tissues. We found that only the correlations between RUNX1 expression and RUNX2 expression were significantly higher among the three genes in ccRCC tissues than in normal tissues. This result suggests that RUNX1 and RUNX2 may jointly exert certain tumor regulatory functions in the development and progression of ccRCC. In addition, immunohistochemical results confirmed the simultaneous high expression of RUNX1 and RUNX2 proteins in ccRCC. While RUNX3 did not show a significant correlation with major ccRCC subtypes, its potential involvement in alternative molecular pathways warrants further investigation. Previous studies suggest that RUNX3 may regulate apoptosis through the TGF‐β and p53 signaling pathways, and its inactivation could contribute to immune evasion and apoptosis resistance in ccRCC [39]. Further research is needed to elucidate the precise mechanisms by which RUNX3 modulates immune regulation and apoptosis, which may provide new insights into its role in ccRCC progression and therapeutic targeting.

Coexpressed genes in tumors often have similar functions or play synergistic biological roles [41]. Coexpression analysis showed that RUNX1 expression and RUNX2 expression were highly correlated with expression of TGFBI, ADAM12, ETV6, GALNT2, C1R, AXL, and IFI16. Except for AXL, all genes had a significant effect on the prognosis of ccRCC patients. Subsequently, 66 coexpressed genes that were significantly associated with both RUNX1 and RUNX2 in ccRCC were subjected to enrichment analysis. The results showed that the coexpressed genes were mainly enriched in cell differentiation, cell adhesion, focal adhesion, and extracellular matrix receptor interactions, which are functions related to tumor progression and metastasis processes. In addition, immune system processes, immune responses, chemokine signaling pathways, and complement and coagulation cascades, which are related to immune regulation of the tumor microenvironment, were also significantly enriched. To further investigate the biological functions of RUNX1 and RUNX2 in ccRCC development, gene enrichment analysis was performed for DEGs based on high and low RUNX1 or RUNX2 expression. Surprisingly, the results of the enrichment analysis showed that RUNX1 and RUNX2 were mainly enriched in pathways related to immune regulation and inflammatory response, further confirming that RUNX1 and RUNX2 are immune‐related genes.

For solid tumors, the TME is mainly composed of tumor cells, multiple immune cell types (T cells, B cells, NK cells, macrophages, and dendritic cells, etc.), and stromal cells (endothelial cells and fibroblasts) [40]. Increasingly, studies have shown that tumor‐infiltrating immune cells, the primary regulators of the TME, not only have a significant impact on tumor progression but also show potential prognostic value [41]. Analysis of the immune infiltration score of the RUNX1/2 high expression group and that of the low expression group showed that RUNX1 and RUNX2 had a significant effect on immune cell infiltration in ccRCC. According to the “MCPCounter” algorithm, we found that the expression of both RUNX1 and RUNX2 in ccRCC showed an extremely strong positive correlation with the presence of fibroblasts. Studies have shown that tumor‐associated fibroblasts (TAFs), which account for more than 50% of stromal cells, cannot only inhibit the function of immune cells by secreting various cytokines (e.g., TGF‐β, IL‐6, CXCL12) or metabolites (e.g., lactate and pyruvate) to promote tumor development, invasion and metastasis but also prevent the penetration of drugs and immune cells into tumor tissues by secreting large amounts of collagen and fibronectin to solidify the extratumor matrix and form a barrier to drug or therapeutic immune cell penetration [42, 43]. In addition, this algorithm showed that the expression of RUNX1 and RUNX2 also correlates with the presence of tumor‐infiltrating immune cells, such as T cells, B cells, NK cells, and monocytes. Subsequently, the “CIBERSORT” algorithm was used to further evaluate the correlation of RUNX1 and RUNX2 with the presence of different subtypes of the above immune cells. In ccRCC tissues with high RUNX1 expression, there was significantly less infiltration of CD8+ T cells, activated NK cells, M1 macrophages, and dendritic cells mediating the antitumor immune response and significantly more infiltration of Tregs, M2 macrophages, and neutrophils mediating the protumor immune response. Similarly, in tumor tissues with high RUNX2 expression, CD8+ T cells, activated NK cells, and dendritic cells infiltrated less, while M2 macrophages infiltrated more. NK cells are the main intrinsic immune cells targeting tumors. Several studies have shown that infiltrating NK cells in ccRCC represent a key component of the antitumor response and that the level of infiltration is significantly associated with patient survival [44]. In addition, NK cells also infiltrate ccRCC lung metastases, and high NK counts are associated with improved survival [45]. Macrophages are classified into M1 (classical) and M2 (alternative) macrophages according to their function and level of secretion of inflammatory factors. Komohara et al. demonstrated a negative correlation between M2 macrophages and ccRCC survival outcome [46]. In addition, tumor cells are capable of converting macrophages into tumor‐associated macrophages (TAMs), the M2d subtype [47]. TAMs can promote tumor cell proliferation by secreting EGF and PDGF, and they can participate in tumor invasion and metastasis by releasing protumor cell metastasis factors such as MMPs (MMP2 and MMP9) and in the immune escape of tumor cells by producing IL10, PGE2, and TGFβ [48, 49, 50]. Tregs are another important immunosuppressive cell in addition to TAMs. Tregs impede antitumor immune responses through multiple mechanisms. In Tregs, CTLA‐4 interferes with costimulatory signaling by binding to CD80/B7‐1 and CD86/B7‐2 in APCs, thereby inhibiting the costimulatory receptor CD28 in effector T cells [51]. The relationship between RUNX gene expression and immune cell infiltration in ccRCC tissues has rarely been reported. Notably, based on bioinformatics analysis, our study is the first to propose the hypothesis that RUNX1 and RUNX2 can drive the formation of a tumor immunosuppressive microenvironment by regulating the level of immune cell infiltration in ccRCC, which in turn promotes tumor progression. It is possible that high expression of RUNX1 and/or RUNX2 inhibits NK‐cell activation, increases Tregs infiltration, and stimulates macrophage differentiation to M2 or even TAMs and adjuvant TAFs formation, thus accelerating ccRCC progression and resulting in a poor prognosis.

Currently, ICI (anti‐PD1) in combination with another ICI (anti‐CTLA‐4) or vascular endothelial growth factor receptor tyrosine kinase inhibitor (VEGFR‐TKI) has become the first‐line treatment option for patients with moderate and unfavorable risk mccRCC. It is essential to note that the loss of functional VHL protein is a hallmark event in the pathogenesis of ccRCC. This loss leads to the accumulation of hypoxia‐inducible factor 2 alpha (HIF‐2α), which, through constitutive activation, plays a central role in driving the oncogenic processes in ccRCC. Belzutifan, a potent small‐molecule inhibitor of HIF‐2α, prevents its heterodimerization with HIF‐1β, thereby inhibiting the formation of an active transcription factor. Clinical trials have demonstrated the efficacy of belzutifan in ccRCC, showing significant benefits over everolimus in terms of progression‐free survival and objective response in patients with advanced ccRCC who had previously received immune checkpoint and antiangiogenic therapies [52]. However, the long‐term response rate to immunotherapy remains relatively low, and resistance is a common challenge. In addition, recent research has highlighted the downregulation of lactotransferrin, a key protein in the innate immune system, as a factor that promotes metastasis in RCC. Interestingly, this downregulation also appears to increase the responsiveness of RCC tumor cells to mTOR inhibitors, suggesting that lactotransferrin levels may serve as a potential predictor of therapeutic effectiveness [53]. Thus, reliable predictive biomarkers are urgently needed to guide immunotherapy for mccRCC patients. In the immunotherapy of ccRCC patients, PD‐1 and CTLA‐4 are the most common and well‐studied blockade targets. In addition, recent studies have revealed that several emerging immune checkpoints, such as CSF1R, LAG3, and TIGIT, have roles in mediating resistance to anti‐pd1 and anti‐ctla4 treatments [54]. In addition, exosomes have emerged as a promising source of noninvasive tumor biomarkers. The unique bilayer membrane structure of exosomes provides protection against external RNases and proteases, thereby enhancing the stability of the mRNAs, miRNAs, and functional proteins they carry. This makes exosomes highly sensitive markers for disease diagnosis. Moreover, the cargo contained within tumor‐derived exosomes, including a range of miRNAs, can serve as valuable biomarkers for RCC detectable in the serum and urine of patients. These exosomal biomarkers hold significant potential for early detection and monitoring of the disease [55]. CSF1R is a secreted cytokine that promotes the transformation of M1 macrophages to TAMs [56]; LAG‐3 is mainly expressed on activated T cells, NK cells, and dendritic cells and not only downregulates T‐cell activity by binding to MHC II molecules but also enhances the suppressive activity of Tregs [57]. TIGIT is mainly present on TILs and exerts tumor immunosuppressive functions mainly by disrupting interleukin production and antigen‐presenting cell (APC) maturation [58]. Currently, there are several clinical trials based on the blockade of the above three immune checkpoints in mccRCC patient immunotherapy in progress. By analyzing the correlation of RUNX1 and RUNX2 expression with that of 19 common immune checkpoint genes involved in ccRCC, we found that RUNX1 and RUNX2 expression were significantly associated with the expression of multiple immune checkpoint genes. Notably, CSF1R, CTLA4, LAG3, and TIGIL expression was particularly elevated in tumor tissues with high expression of RUNX1 or RUNX2. Based on data from the IMvigor210 cohort, the assessment of the impact of RUNX1 and RUNX2 expression on the response to immunotherapy and its impact on the prognosis of the patients in the cohort was analyzed. Although the patients in this cohort were uroepithelial carcinoma patients, according to the results and combined with the immune checkpoint correlation analysis, we can infer that RUNX1 has the potential to serve as a marker for the degree of response to immunotherapy in ccRCC patients, but further confirmation is pending in the ccRCC immunotherapy cohort.

The clinical trial NCT06090669 explores the use of imatinib to modulate RUNX1 activity in patients with germline RUNX1 mutations associated with hematologic malignancies. Given that RUNX1 is involved in tumor progression and immune regulation in ccRCC, this trial highlights the potential therapeutic relevance of targeting RUNX1. Since imatinib inhibits ABL‐mediated phosphorylation of RUNX1, restoring its function, similar approaches could be investigated in solid tumors like ccRCC. Our findings suggest that RUNX1 plays a crucial role in ccRCC prognosis and immune infiltration, warranting further research into RUNX1‐targeted therapies for improving patient outcomes.

Prognostic analysis, functional prediction, and immune correlation assessment revealed that RUNX1 and RUNX2 are key IIRGs in ccRCC progression and that their expression correlates with patient prognosis. To further assess the feasibility of RUNX1 or RUNX2 as independent prognostic biomarkers, a multivariate Cox regression model was developed. The results showed that RUNX1 could be used as an independent prognostic indicator, while RUNX2 could not. However, it was not accurate enough to consider only a single prognostic biomarker or an individual clinicopathological characteristic to predict patient prognosis. Therefore, we combined RUNX1 expression with several clinicopathological characteristics to construct a nomogram to evaluate the prognosis of ccRCC patients. The nomogram was confirmed by ROC curves to have the highest accuracy in predicting the prognosis of ccRCC.

There are some limitations in this study. While our research provides valuable insights into the roles of RUNX1 and RUNX2 in ccRCC, certain limitations should be acknowledged. Although we conducted a preliminary exploration of the potential molecular pathways involving RUNX1 and RUNX2, a more comprehensive analysis, including protein‐protein interaction studies and co‐expression network analysis, is necessary to further elucidate their regulatory mechanisms. In addition, while our bioinformatics findings suggest a strong association between RUNX1/2 and immune modulation, experimental validation through RT‐qPCR and functional assays is needed to confirm these observations. Moreover, our study was primarily based on bioinformatics analysis using publicly available data sets, which may introduce potential biases. The precise mechanisms through which RUNX1 and RUNX2 regulate the immune microenvironment in ccRCC remain unclear and require further experimental investigation. Another limitation is that our immunotherapy response analysis was conducted using clinical data from patients with uroepithelial carcinoma, and the impact of RUNX1 and RUNX2 expression on immunotherapy response in ccRCC needs further evaluation in dedicated immunotherapy cohorts. In addition, the RUNX1‐based nomogram, while demonstrating promising predictive value, has yet to be validated in a large, independent clinical cohort. Furthermore, potential confounding factors such as tumor heterogeneity, patient treatment history, and tumor microenvironment alterations may influence the observed associations between RUNX1/2 expression and immune modulation in ccRCC. While our study provides important insights, future research incorporating single‐cell transcriptomics and longitudinal patient data will be necessary to address these variables and validate our findings.

## 5 Conclusions

In conclusion, we demonstrated that all RUNX family members are overexpressed in ccRCC, with RUNX1 and RUNX2 expression significantly correlated with several clinicopathological parameters and prognosis of patients. The correlation between RUNX1 expression and RUNX2 expression was significantly higher in ccRCC tumor tissues, suggesting a possible cluster‐regulatory role. RUNX1 and RUNX2 are important IIRGs, and their high expression in tumor tissues is closely associated with an increased proportion of TAFs, elevated levels of infiltration of multiple immunosuppressive cells, and upregulated expression of key immune checkpoint genes. Our study reveals the potential value of RUNX1 and RUNX2 in ccRCC development and immune microenvironment regulation, as well as the promise of RUNX1 as a biomarker of immunotherapeutic response. In addition, based on the confirmation that RUNX1 can be used as an independent prognostic marker for ccRCC, we further developed a new RUNX1‐based nomogram to estimate the survival outcome of ccRCC patients by combining several clinicopathological parameters, which allows for a better model for the evaluation of patient prognosis prediction.

## Author Contributions

**Jingang Jian:** conceptualization, data curation, formal analysis, funding acquisition, investigation, methodology, project administration, resources, software, supervision, validation, visualization, writing – original draft, writing – review and editing. **Jun Zhang:** conceptualization, data curation, resources, software, validation, writing – original draft, writing – review and editing. **Hongbo Xu:** conceptualization, data curation, formal analysis, methodology, software, visualization, writing – original draft, writing – review and editing. **He Wang:** conceptualization, data curation, formal analysis, funding acquisition, investigation, methodology, project administration, resources, software, supervision, validation, visualization, writing – original draft, writing – review and editing. **Jianquan Hou:** conceptualization, funding acquisition, investigation, project administration, resources, supervision, visualization, writing – review and editing.

## Ethics Statement

The authors have nothing to report.

## Conflicts of Interest

The authors declare no conflicts of interest.

## Supporting information


**Figure S1:** Expression levels of RUNX1/2/3 in different subgroups of ccRCC.

**Figure S2:** Effect of RUNX1/2/3 expression levels on disease‐free survival of ccRCC patients.

**Figure S3:** GO (MF) enrichment analysis.

**Figure S4:** RUNX1 and RUNX2 expression correlated with immune checkpoints in ccRCC samples.

**Figure S5:** Original images of Gels and Blots.

**Supplementary material**.

## Data Availability

The authors have nothing to report.

## References

[1] R. L. Siegel , A. N. Giaquinto , and A. Jemal , “Cancer Statistics, 2024,” CA: A Cancer Journal for Clinicians 74, no. 1 (2024): 12–49, 10.3322/caac.21820. 38230766

[2] E. Rassy , P. Parent , F. Lefort , S. Boussios , G. Baciarello , and N. Pavlidis , “New Rising Entities in Cancer of Unknown Primary: Is There a Real Therapeutic Benefit?,” Critical Reviews in Oncology/Hematology 147 (2020): 102882, 10.1016/j.critrevonc.2020.102882. 32106012

[3] U. Capitanio , K. Bensalah , A. Bex , et al., “Epidemiology of Renal Cell Carcinoma,” European Urology 75, no. 1 (2019): 74–84, 10.1016/j.eururo.2018.08.036. PMC8397918 30243799

[4] J. Tran and M. C. Ornstein , “Clinical Review on the Management of Metastatic Renal Cell Carcinoma,” JCO Oncology Practice 18, no. 3 (2022): 187–196, 10.1200/OP.21.00419. 34529499

[5] N. Borcherding , A. Vishwakarma , A. P. Voigt , et al., “Mapping the Immune Environment in Clear Cell Renal Carcinoma by Single‐Cell Genomics,” Communications Biology 4, no. 1 (2021): 122, 10.1038/s42003-020-01625-6. PMC7840906 33504936

[6] L. Vuong , R. R. Kotecha , M. H. Voss , and A. A. Hakimi , “Tumor Microenvironment Dynamics in Clear‐Cell Renal Cell Carcinoma,” Cancer Discovery 9, no. 10 (2019): 1349–1357, 10.1158/2159-8290.CD-19-0499. PMC6774890 31527133

[7] I. Y. Sheng and M. C. Ornstein , “Ipilimumab and Nivolumab as First‐Line Treatment of Patients With Renal Cell Carcinoma: The Evidence to Date,” Cancer Management and Research 12 (2020): 4871–4881, 10.2147/CMAR.S202017. PMC7320748 32606975

[8] J. E. Brown , K. L. Royle , W. Gregory , et al., “Temporary Treatment Cessation Versus Continuation of First‐Line Tyrosine Kinase Inhibitor in Patients With Advanced Clear Cell Renal Cell Carcinoma (STAR): An Open‐Label, Non‐Inferiority, Randomised, Controlled, Phase 2/3 Trial,” Lancet Oncology 24, no. 3 (2023): 213–227, 10.1016/S1470-2045(22)00793-8. 36796394

[9] D. Miao , J. Shi , Z. Xiong , et al., “As a Prognostic Biomarker of Clear Cell Renal Cell Carcinoma RUFY4 Predicts Immunotherapy Responsiveness in a PDL1‐Related Manner,” Cancer Cell International 22 (2022): 66, 10.1186/s12935-022-02480-7. PMC8822671 35135552

[10] K. Bi , M. X. He , Z. Bakouny , et al., “Tumor and Immune Reprogramming During Immunotherapy in Advanced Renal Cell Carcinoma,” Cancer Cell 39, no. 5 (2021): 649–661.e5, 10.1016/j.ccell.2021.02.015. PMC8115394 33711272

[11] B. Otalora‑Otalora , B. Henriquez , L. Lopez‑Kleine , and A. Rojas , “RUNX Family: Oncogenes or Tumor Suppressors (Review),” Oncology Reports 42, no. 1 (2019): 3–19, 10.3892/or.2019.7149. PMC6549079 31059069

[12] R. Sood , Y. Kamikubo , and P. Liu , “Role of RUNX1 in Hematological Malignancies,” Blood 129, no. 15 (2017): 2070–2082, 10.1182/blood-2016-10-687830. PMC5391618 28179279

[13] Y. Ito , S. C. Bae , and L. S. H. Chuang , “The RUNX Family: Developmental Regulators in Cancer,” Nature Reviews Cancer 15, no. 2 (2015): 81–95, 10.1038/nrc3877. 25592647

[14] Q. Li , Q. Lai , C. He , et al., “RUNX1 Promotes Tumour Metastasis by Activating the Wnt/β‐Catenin Signalling Pathway and EMT in Colorectal Cancer,” Journal of Experimental & Clinical Cancer Research 38, no. 1 (2019): 334, 10.1186/s13046-019-1330-9. PMC6670220 31370857

[15] C. S. L. Hoi , S. E. Lee , S. Y. Lu , et al., “Runx1 Directly Promotes Proliferation of Hair Follicle Stem Cells and Epithelial Tumor Formation in Mouse Skin,” Molecular and Cellular Biology 30, no. 10 (2010): 2518–2536, 10.1128/MCB.01308-09. PMC2863705 20308320

[16] C. Y. Wu , L. Li , S. L. Chen , X. Yang , C. Z. Zhang , and Y. Cao , “A Zic2/Runx2/NOLC1 Signaling Axis Mediates Tumor Growth and Metastasis in Clear Cell Renal Cell Carcinoma,” Cell Death & Disease 12, no. 4 (2021): 319, 10.1038/s41419-021-03617-8. PMC7994417 33767130

[17] D. T. Leong , J. Lim , X. Goh , et al., “Cancer‐Related Ectopic Expression of the Bone‐Related Transcription Factor RUNX2 in Non‐Osseous Metastatic Tumor Cells Is Linked to Cell Proliferation and Motility,” Breast Cancer Research 12, no. 5 (2010): R89, 10.1186/bcr2762. PMC3096982 21029421

[18] T. Tsunematsu , Y. Kudo , S. Iizuka , et al., “RUNX3 Has an Oncogenic Role in Head and Neck Cancer,” PLoS ONE 4, no. 6 (2009): e5892, 10.1371/journal.pone.0005892. PMC2690822 19521519

[19] K. Gao , F. Zhang , K. Chen , et al., “Expression Patterns and Prognostic Value of RUNX Genes in Kidney Cancer,” Scientific Reports 11, no. 1 (2021): 14934, 10.1038/s41598-021-94294-2. PMC8298387 34294773

[20] N. Rooney , S. M. Mason , L. McDonald , et al., “RUNX1 Is a Driver of Renal Cell Carcinoma Correlating With Clinical Outcome,” Cancer Research 80, no. 11 (2020): 2325–2339, 10.1158/0008-5472.CAN-19-3870. 32156779

[21] T. Li , J. Fan , B. Wang , et al., “TIMER: A Web Server for Comprehensive Analysis of Tumor‐Infiltrating Immune Cells,” Cancer Research 77, no. 21 (2017): e108–e110, 10.1158/0008-5472.CAN-17-0307. PMC6042652 29092952

[22] A. Colaprico , T. C. Silva , C. Olsen , et al., “TCGAbiolinks: An R/Bioconductor Package for Integrative Analysis of TCGA Data,” Nucleic Acids Research 44, no. 8 (2016): e71, 10.1093/nar/gkv1507. PMC4856967 26704973

[23] R. Edgar , “Gene Expression Omnibus: NCBI Gene Expression and Hybridization Array Data Repository,” Nucleic Acids Research 30, no. 1 (2002): 207–210, 10.1093/nar/30.1.207. PMC99122 11752295

[24] M. E. Ritchie , B. Phipson , D. Wu , et al., “Limma Powers Differential Expression Analyses for RNA‐Sequencing and Microarray Studies,” Nucleic Acids Research 43, no. 7 (2015): e47, 10.1093/nar/gkv007. PMC4402510 25605792

[25] D. S. Chandrashekar , B. Bashel , S. A. H. Balasubramanya , et al., “UALCAN: A Portal for Facilitating Tumor Subgroup Gene Expression and Survival Analyses,” Neoplasia 19, no. 8 (2017): 649–658, 10.1016/j.neo.2017.05.002. PMC5516091 28732212

[26] A. Asplund , P. H. D. Edqvist , J. M. Schwenk , and F. Pontén , “Antibodies for Profiling the Human Proteome—The Human Protein Atlas as a Resource for Cancer Research,” Proteomics 12, no. 13 (2012): 2067–2077, 10.1002/pmic.201100504. 22623277

[27] Z. Tang , C. Li , B. Kang , G. Gao , C. Li , and Z. Zhang , “GEPIA: A Web Server for Cancer and Normal Gene Expression Profiling and Interactive Analyses,” Nucleic Acids Research 45, no. W1 (2017): W98–W102, 10.1093/nar/gkx247. PMC5570223 28407145

[28] E. Cerami , J. Gao , U. Dogrusoz , et al., “The cBio Cancer Genomics Portal: An Open Platform for Exploring Multidimensional Cancer Genomics Data,” Cancer Discovery 2, no. 5 (2012): 401–404, 10.1158/2159-8290.CD-12-0095. PMC3956037 22588877

[29] G. Yu , L. G. Wang , Y. Han , and Q. Y. He , “clusterProfiler: An R Package for Comparing Biological Themes Among Gene Clusters,” OMICS: A Journal of Integrative Biology 16, no. 5 (2012): 284–287, 10.1089/omi.2011.0118. PMC3339379 22455463

[30] M. D. Robinson , D. J. McCarthy , and G. K. Smyth , “edgeR: A Bioconductor Package for Differential Expression Analysis of Digital Gene Expression Data,” Bioinformatics 26, no. 1 (2010): 139–140, 10.1093/bioinformatics/btp616. PMC2796818 19910308

[31] E. Becht , N. A. Giraldo , L. Lacroix , et al., “Estimating the Population Abundance of Tissue‐Infiltrating Immune and Stromal Cell Populations Using Gene Expression,” Genome Biology 17, no. 1 (2016): 218, 10.1186/s13059-016-1070-5. PMC5073889 27765066

[32] B. Chen , M. S. Khodadoust , C. L. Liu , A. M. Newman , and A. A. Alizadeh , “Profiling Tumor Infiltrating Immune Cells With CIBERSORT,” in Cancer Systems Biology. Methods in Molecular Biology, ed. L. Von Stechow . Springer New York, 2018), 243–259, 10.1007/978-1-4939-7493-1_12. PMC5895181 29344893

[33] A. V. Balar , M. D. Galsky , J. E. Rosenberg , et al., “Atezolizumab as First‐Line Treatment in Cisplatin‐Ineligible Patients With Locally Advanced and Metastatic Urothelial Carcinoma: A Single‐Arm, Multicentre, Phase 2 Trial,” Lancet 389, no. 10064 (2017): 67–76, 10.1016/S0140-6736(16)32455-2. PMC5568632 27939400

[34] F. Li , M. Yang , Y. Li , et al., “An Improved Clear Cell Renal Cell Carcinoma Stage Prediction Model Based on Gene Sets,” BMC Bioinformatics 21, no. 1 (2020): 232, 10.1186/s12859-020-03543-0. PMC7278205 32513106

[35] T. Tang , X. Huang , G. Zhang , Z. Hong , X. Bai , and T. Liang , “Advantages of Targeting the Tumor Immune Microenvironment Over Blocking Immune Checkpoint in Cancer Immunotherapy,” Signal Transduction and Targeted Therapy 6, no. 1 (2021): 72, 10.1038/s41392-020-00449-4. PMC7896069 33608497

[36] E. Jonasch , C. L. Walker , and W. K. Rathmell , “Clear Cell Renal Cell Carcinoma Ontogeny and Mechanisms of Lethality,” Nature Reviews Nephrology 17, no. 4 (2021): 245–261, 10.1038/s41581-020-00359-2. PMC8172121 33144689

[37] K. Ueda , S. Suekane , H. Kurose , et al., “Prognostic Value of PD‐1 and PD‐L1 Expression in Patients With Metastatic Clear Cell Renal Cell Carcinoma,” Urologic Oncology: Seminars and Original Investigations 36, no. 11 (2018): 499.e9–499.e16, 10.1016/j.urolonc.2018.07.003. 30131293

[38] S. Mikami , R. Mizuno , T. Kondo , et al., “Clinical Significance of Programmed Death‐1 and Programmed Death‐Ligand 1 Expression in the Tumor Microenvironment of Clear Cell Renal Cell Carcinoma,” Cancer Science 110, no. 6 (2019): 1820–1828, 10.1111/cas.14019. PMC6550131 30972888

[39] F. Chen , X. Liu , Q. Cheng , S. Zhu , J. Bai , and J. Zheng , “RUNX3 Regulates Renal Cell Carcinoma Metastasis via Targeting miR‐6780a‐5p/E‐Cadherin/EMT Signaling Axis,” Oncotarget 8, no. 60 (2017): 101042–101056, 10.18632/oncotarget.13205. PMC5731854 29254144

[40] A. Sathe , S. M. Grimes , B. T. Lau , et al., “Single‐Cell Genomic Characterization Reveals the Cellular Reprogramming of the Gastric Tumor Microenvironment,” Clinical Cancer Research 26, no. 11 (2020): 2640–2653, 10.1158/1078-0432.CCR-19-3231. PMC7269843 32060101

[41] S. Liu , S. Li , Y. Wang , et al., “Prognostic Value of Infiltrating Immune Cells in Clear Cell Renal Cell Carcinoma (ccRCC),” Journal of Cellular Biochemistry 121, no. 3 (2020): 2571–2581, 10.1002/jcb.29479. 31823423

[42] M. E. Fiori , S. Di Franco , L. Villanova , P. Bianca , G. Stassi , and R. De Maria , “Cancer‐Associated Fibroblasts as Abettors of Tumor Progression at the Crossroads of EMT and Therapy Resistance,” Molecular Cancer 18, no. 1 (2019): 70, 10.1186/s12943-019-0994-2. PMC6441236 30927908

[43] F. Wu , J. Yang , J. Liu , et al., “Signaling Pathways in Cancer‐Associated Fibroblasts and Targeted Therapy for Cancer,” Signal Transduction and Targeted Therapy 6, no. 1 (2021): 218, 10.1038/s41392-021-00641-0. PMC8190181 34108441

[44] I. Terrén , A. Orrantia , I. Mikelez‐Alonso , J. Vitallé , O. Zenarruzabeitia , and F. Borrego , “NK Cell‐Based Immunotherapy in Renal Cell Carcinoma,” Cancers 12, no. 2 (2020): 316, 10.3390/cancers12020316. PMC7072691 32013092

[45] R. Remark , M. Alifano , I. Cremer , et al., “Characteristics and Clinical Impacts of the Immune Environments in Colorectal and Renal Cell Carcinoma Lung Metastases: Influence of Tumor Origin,” Clinical Cancer Research 19, no. 15 (2013): 4079–4091, 10.1158/1078-0432.CCR-12-3847. 23785047

[46] Y. Komohara , H. Hasita , K. Ohnishi , et al., “Macrophage Infiltration and Its Prognostic Relevance in Clear Cell Renal Cell Carcinoma,” Cancer Science 102, no. 7 (2011): 1424–1431, 10.1111/j.1349-7006.2011.01945.x. 21453387

[47] Y. Chen , Y. Song , W. Du , L. Gong , H. Chang , and Z. Zou , “Tumor‐Associated Macrophages: An Accomplice in Solid Tumor Progression,” Journal of Biomedical Science 26, no. 1 (2019): 78, 10.1186/s12929-019-0568-z. PMC6800990 31629410

[48] Y. Lin , J. Xu , and H. Lan , “Tumor‐Associated Macrophages in Tumor Metastasis: Biological Roles and Clinical Therapeutic Applications,” Journal of Hematology & Oncology 12, no. 1 (2019): 76, 10.1186/s13045-019-0760-3. PMC6626377 31300030

[49] S. Quintero‐Fabián , R. Arreola , E. Becerril‐Villanueva , et al., “Role of Matrix Metalloproteinases in Angiogenesis and Cancer,” Frontiers in Oncology 9 (2019): 1370, 10.3389/fonc.2019.01370. PMC6915110 31921634

[50] J. Zhou , Z. Tang , S. Gao , C. Li , Y. Feng , and X. Zhou , “Tumor‐Associated Macrophages: Recent Insights and Therapies,” Frontiers in Oncology 10 (2020): 188, 10.3389/fonc.2020.00188. PMC7052362 32161718

[51] N. Beyersdorf , T. Kerkau , and T. Hünig , “CD28 Co‐Stimulation in T‐Cell Homeostasis: A Recent Perspective,” ImmunoTargets and Therapy 4 (2015): 111, 10.2147/ITT.S61647. PMC4918251 27471717

[52] T. K. Choueiri , T. Powles , K. Peltola , et al., “Belzutifan Versus Everolimus for Advanced Renal‐Cell Carcinoma,” New England Journal of Medicine 391, no. 8 (2024): 710–721, 10.1056/NEJMoa2313906. 39167807

[53] H. Aweys , D. Lewis , M. Sheriff , et al., “Renal Cell Cancer – Insights in Drug Resistance Mechanisms,” Anticancer Research 43, no. 11 (2023): 4781–4792, 10.21873/anticanres.16675. 37909991

[54] K. Takamatsu , N. Tanaka , K. Hakozaki , et al., “Profiling the Inhibitory Receptors LAG‐3, TIM‐3, and TIGIT in Renal Cell Carcinoma Reveals Malignancy,” Nature Communications 12, no. 1 (2021): 5547, 10.1038/s41467-021-25865-0. PMC8452744 34545095

[55] S. Boussios , P. Devo , I. C. A. Goodall , et al., “Exosomes in the Diagnosis and Treatment of Renal Cell Cancer,” International Journal of Molecular Sciences 24, no. 18 (2023): 14356, 10.3390/ijms241814356. PMC10531522 37762660

[56] Y. Zhu , B. L. Knolhoff , M. A. Meyer , et al., “CSF1/CSF1R Blockade Reprograms Tumor‐Infiltrating Macrophages and Improves Response to T‐Cell Checkpoint Immunotherapy in Pancreatic Cancer Models,” Cancer Research 74, no. 18 (2014): 5057–5069, 10.1158/0008-5472.CAN-13-3723. PMC4182950 25082815

[57] C. T. Huang , C. J. Workman , D. Flies , et al., “Role of LAG‐3 in Regulatory T Cells,” Immunity 21, no. 4 (2004): 503–513, 10.1016/j.immuni.2004.08.010. 15485628

[58] A. C. Anderson , N. Joller , and V. K. Kuchroo , “Lag‐3, Tim‐3, and TIGIT: Co‐Inhibitory Receptors With Specialized Functions in Immune Regulation,” Immunity 44, no. 5 (2016): 989–1004, 10.1016/j.immuni.2016.05.001. PMC4942846 27192565

