# Supplementary material for: RUNX1 Is an Independent Prognostic Marker Involved in Immune Infiltration and Therapeutic Responses in Clear Cell Renal Cell Carcinoma Patients Through Cluster Regulation With RUNX2
Source: Immun Inflamm Dis. 2025 Aug 14;13(8):e70242. doi: 10.1002/iid3.70242 (PMC13757348; doi:10.1002/iid3.70242)
Supplement: Supplementary file 1 [file IID3-13-e70242-s004.docx]

**Supplementary material**

**Figure. S1.** Expression levels of RUNX1/2/3 in different subgroups of ccRCC. Analysis of RUNX1**(A)**, RUNX2**(B)**, and RUNX3**(C)** expression levels in normal tissues and ccRCC subgroups (ccA and ccB, respectively).

**Figure. S2.** Effect of RUNX1/2/3 expression levels on disease-free survival of ccRCC patients.

Investigation of the effect of RUNX1 **(A)**, RUNX2**(B)**, and RUNX3 **(C)** expression levels on disease-free survival of ccRCC patients by GEPIA online analysis.

**Figure. S3.** GO (MF) enrichment analysis. MF enrichment entries of differentially expressed genes between high and low RUNX1 **(A)** or RUNX2 **(B)** mRNA expression groups based on gseGO.

**Figure. S4.** RUNX1 and RUNX2 expression correlated with immune checkpoints in ccRCC samples. RUNX1and RUNX2 significantly correlated with PDCD1 (**A**, **F**), CTLA-4 (**B**, **G**), LAG3 (**C**, **H**), TIGIT (**D**, **I**) and BTLA(**E**,**J**), respeetively.

**Figure. S5.** Original images of Gels and Blots.
